# Sepsis-Inducing Leukocytoclastic Vasculitis Resistant to Steroid Therapy: A Case Report

**DOI:** 10.7759/cureus.70051

**Published:** 2024-09-23

**Authors:** Jena Jacobs, Joseph Norman, Stephen Verral

**Affiliations:** 1 Dermatology, A.T. Still University, Kirksville, USA; 2 Otolaryngology, A.T. Still University, Kirksville, USA; 3 Dermatology, Gateway Dermatology, Glens Falls, USA

**Keywords:** hemorrhagic bullae, idiopathic leukocytoclastic vasculitis, leukocytoclastic vasculitis (lcv), palpable purpura, sepsis

## Abstract

A 34-year-old White male presented with a persistent rash on the lower extremities characterized by erythema, liquid drainage, and severe burning pain. Initially misattributed to contact dermatitis, the condition worsened despite treatment with prednisone and doxycycline. Leukocytoclastic vasculitis (LCV) was confirmed via punch biopsy. The patient’s treatment involved conservative measures, systemic prednisone therapy, doxycycline, and later adjunctive dapsone. Nevertheless, the patient developed secondary bacterial infection with methicillin-sensitive *Staphylococcus aureus* and *Pseudomonas aeruginosa*. This case highlights an uncommon presentation of idiopathic LCV that led to sepsis and reviews management for persistent vasculitis.

## Introduction

Leukocytoclastic vasculitis (LCV) is an inflammatory condition affecting the small vessels of the dermal capillaries and venules [1]. It often presents acutely as bilateral palpable purpura on the lower extremities. The condition may be idiopathic or linked to malignancies, medications, autoimmune disorders, and infections [2]. Most cases are confined to the skin and resolve with conservative treatment [3]. It can also be associated with systemic involvement requiring further treatment, such as oral corticosteroids. In some cases, the diagnosis of LCV is one of exclusion [4]. Histopathological diagnosis is key and typically shows neutrophilic infiltration surrounding the vessel wall with signs of leukocytoclasia and fibrinoid necrosis [5]. We present the case of a 34-year-old male who developed idiopathic hemorrhagic-bullous LCV that did not improve with systemic steroid therapy, leading to hospitalization for sepsis, secondary to his lesions.

## Case presentation

A 34-year-old White male patient presented to the dermatology clinic with an extremely painful rash on his lower extremities initially thought to be caused by poison ivy. The rash began two weeks earlier as petechiae and progressed to vesicles that burst, draining serous fluid. He had previously been seen at an urgent care center, where he was prescribed 40 mg of prednisone and 100 mg of doxycycline. His medical history includes hypogonadotropic hypogonadism, a prior splenectomy, and a previous episode of deep vein thrombosis (DVT). The DVT is thought to be provoked by a combination of an ankle fracture and immobilization from a boot that occurred in 7/2022. He has been on daily rivaroxaban since that date due to an occlusive progressing thrombus in the distal femoral vein. His other daily medications are tamoxifen and testosterone cypionate.

**Figure 1:**
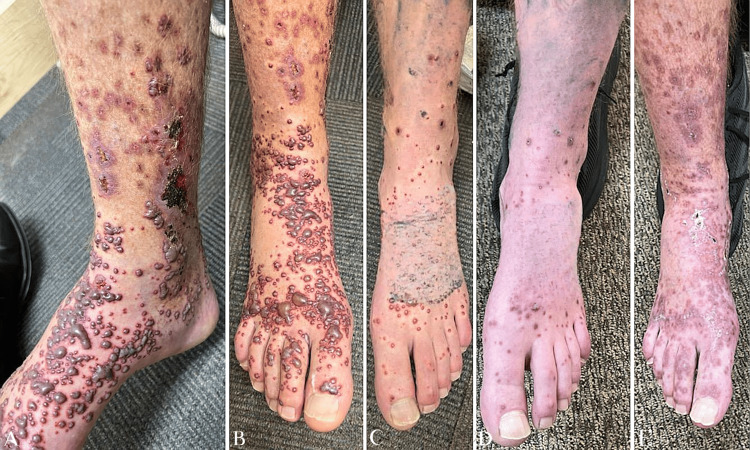
Right and left legs before treatment (A, B, and C) and after treatment (D and E). Hemorrhagic bullae have resolved into coalescing hyperpigmented plaques with some overlying crust.

The patient denied any recent changes to his longstanding medications and reported no known triggering events prior to the onset of the rash. He also stated that he had never experienced similar symptoms before. The patient denied a history of MRSA (methicillin-resistant *Staphylococcus aureus*), recent travel outside the country, cold sores, recent insect bites, or known sick contacts. He did report a COVID-19 infection one year before the rash appeared. There were no systemic symptoms, and the review of systems was negative except for the skin changes. On examination, the skin showed hemorrhagic well-circumscribed tense vesicles and bullae coalescing into patches with background erythema on both lower extremities. Most of these lesions were clustered over the anterior tibial region and the dorsum of the foot (Figures 1A-1C).

Given the high suspicion of vasculitis, a punch biopsy was performed in the office. Blood tests were also conducted to investigate potential hematologic, rheumatic, and infectious causes. Notably, results showed leukocytosis (13,700 cells/μL), elevated liver enzymes with alanine aminotransferase (108 U/L) and aspartate aminotransferase (56 U/L), hypercalcemia (10.4 mg/dL), and a slightly elevated prothrombin time/international normalized ratio (INR) (14.2 seconds, 1.3). All other values were within normal limits (Table 1). The patient continued treatment with doxycycline 100 mg twice daily, prednisone 40 mg once daily, and the addition of compression stockings.

**Table 1:** Pertinent laboratory results ELISA: enzyme-linked immunosorbent assay; SSA: Sjögren’s syndrome-A; SSB: Sjögren’s syndrome-B; µL: microliter; mg: milligram; dL: deciliter; IU: international units; U/L: units per liter; mm/h: millimeter per hour; IgG: immunoglobulin G; IgM: immunoglobulin M

Laboratory Tests	Patient's Value	Normal Range
White Blood Cell Count	13,700/µL	1,800-7,400/µL
Red Blood Cell Count	5.49 million/µL	4.35 to 5.65 million/µL
Calcium	10.4 mg/dL	8.6-10.2 mg/dL
Alanine Aminotransferase	108 U/L	<66 U/L
Aspartate Aminotransferase	56 U/L	<44 U/L
Creatinine	0.89 mg/dL	0.6-1.2 mg/dL
Blood Urea Nitrogen	15.2 mg/dL	6-20 mg/dL
Albumin	4.5 g/dL	3.5-5.2 g/dL
C-reactive Protein	<0.30 mg/dL	<0.50 mg/dL
Varicella Zoster Virus IgM	Negative	Negative
Varicella Zoster Virus IgG	Positive	Negative
Glucose-6-Phosphate Dehydrogenase	15.9 units/g hemoglobin	7-20.5 units/g hemoglobin
Rheumatoid Factor	<10 IU/mL	<13.9 IU/mL
Cyclic Citrullinated Peptide Antibody	5 units	<20 units
SSA/SSB Sjogren's Antibodies	Negative	Negative
Myeloma Spike Serum Immunoglobins	Not observed	Not observed
CH-50 Total Complement Activity	57 U/mL	>41 U/mL
Activated Partial Thromboplastin Time	32.7 seconds	25.1-36.5 seconds
Protime	14.2 seconds	9.4-12.5 seconds
International Normalized Ratio (INR)	1.3	0.8-1.2
Indirect Immunofluorescence for Anti-neutrophil Cytoplasmic Antibody	Negative	Negative
ELISA Antibodies to Proteinase 3 and Myeloperoxidase	Negative	Negative
Immunoglobulin G	833 mg/dL	614-1,295 mg/dL
Immunoglobulin A	351	69-309
Immunoglobulin M	110	53-334
Urinalysis	Negative	Negative
Erythrocyte Sedimentation Rate	6 mm/h	0-14 mm/h
Varicella Zoster Antibody - IgG	233	Immune>165
Varicella Zoster Antibody - IgM	<0.91	0.00-0.90
Wound Culture	3+ *Pseudomonas aeruginosa*, 3+* Staphylococcus aureus*	No microorganisms

**Figure 2:**
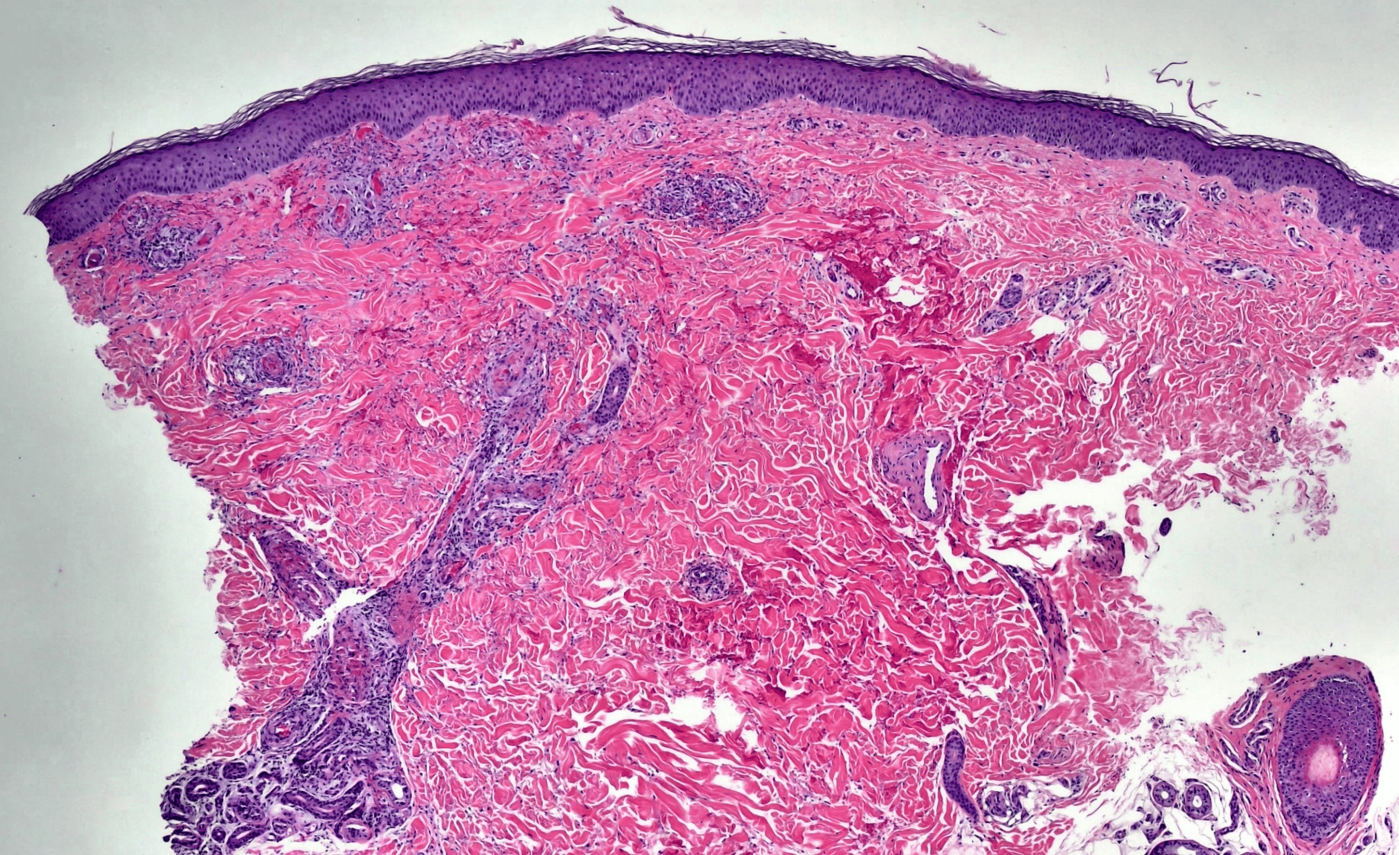
Punch biopsy submitted in formalin measuring 4x4x5 mm (4X). There is superficial and deep perivascular inflammatory infiltrate composed of lymphocytes, histiocytes, neutrophils, and eosinophils. Extravasated erythrocytes and nuclear dust are present in the dermis. There is fibrinoid necrosis of the vessels.

**Figure 3:**
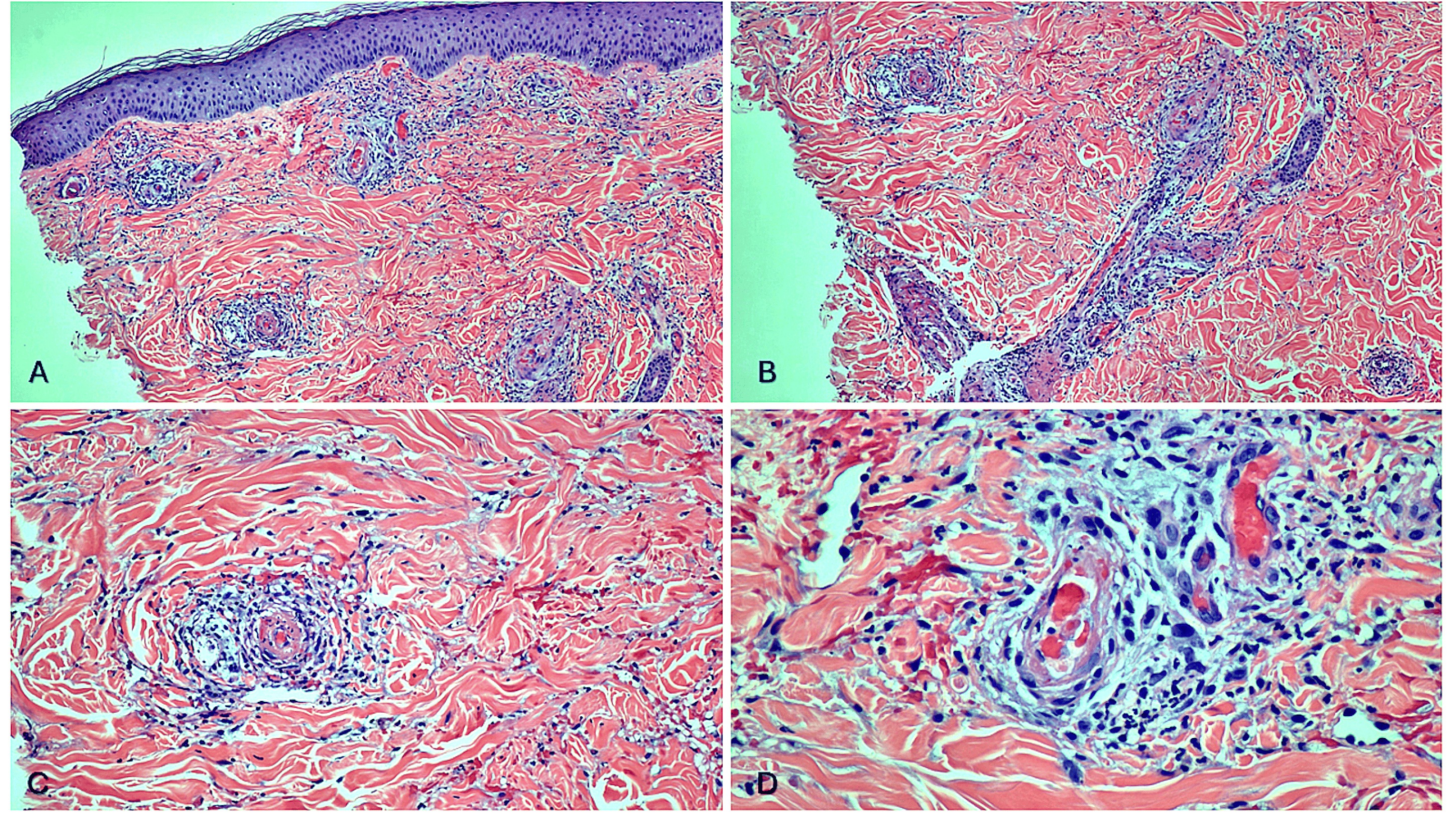
There is superficial and deep perivascular inflammatory infiltrate composed of lymphocytes, histiocytes, neutrophils, and eosinophils. Extravasated erythrocytes and nuclear dust are present in the dermis. There is fibrinoid necrosis of the vessels (A & B: 10X; C: 20X; D: 40X).

At a follow-up appointment one week later, the rash had worsened, spreading to the thighs and abdomen. The biopsy confirmed LCV (Figure 2 and Figures 3A-3D), leading to the initiation of dapsone 50 mg twice daily and 0.05% clobetasol ointment. A glucose-6-phosphate dehydrogenase (G6PD) level was checked before starting dapsone (Table 1). Although his rash began to improve, the patient suddenly deteriorated as he was in 10/10 pain, could not bear weight, and was febrile. He was sent to the emergency room by his primary care physician. He was hospitalized for suspected cellulitis arising from his ulcerated lesions. Blood cultures taken in the emergency department were positive for *S. aureus* and *Pseudomonas aeruginosa*. Resistance testing showed that the *S. aureus* was sensitive to methicillin but resistant to tetracyclines. The addition of methotrexate for vasculitis management was considered; however, after discussing options with the patient, a shared decision was made to continue the existing regimen. The infection was successfully cleared with cefazolin and ciprofloxacin.

After completing antibiotic therapy, the patient was reassessed at the dermatology clinic, showing slight improvement in the skin rash with resolution of the hemorrhagic bullae and vesicles (Figures 1D-1E). The Massachusetts General Vasculitis Center was consulted for further management and evaluation. They repeated the work-up for LCV, including autoimmune blood tests, and a skin biopsy with additional immunofluorescence. All tests came back negative, leading to a diagnosis of idiopathic LCV. The patient was tapered off dapsone, clobetasol, and prednisone over a two-month period.

## Discussion

LCV represents a multifactorial condition with potential triggers spanning pharmacological agents, infections, or malignancies. Among systemic diseases, the strongest associations with LCV include antineutrophil cytoplasmic antibody (ANCA)-associated vasculitides, connective tissue disorders, cryoglobulinemic vasculitis, IgA vasculitis (formerly known as Henoch-Schönlein purpura), and hypocomplementemic urticarial vasculitis (HUV) [5].

In clinical practice, when LCV is suspected, it necessitates an exhaustive diagnostic approach to delineate whether the vasculitic process is confined to the skin or reflects an underlying systemic vasculitis [5]. Standard laboratory investigations typically include assessments of platelet count, renal function, and urinalysis, alongside serological tests for hepatitis B and C, autoantibodies such as anti-nuclear and ANCA, and complement fractions [3]. Notably, IgA staining in biopsy specimens remains a critical diagnostic marker, particularly in distinguishing IgA vasculitis.

Our patient was on two medications, tamoxifen and rivaroxaban, both of which have been associated with cases of LCV [6-8]. However, the patient had been using these medications for several months/years without any previous reactions. Additionally, there has been a reported case of LCV following a COVID-19 infection, which the patient had contracted a year prior [9].

Hemorrhagic bullae, which our patient had, are a less common manifestation of LCV and may indicate the involvement of medium-sized vessels in the vasculitis [6]. These lesions increase the risk of infection due to skin barrier disruption and the formation of ulcerative wounds. To reduce this risk, we continued the patient on a prophylactic regimen of doxycycline while awaiting the resolution of the bullae. Despite this precaution, the patient developed an infection caused by bacteria that were resistant to the prescribed antibiotic.

Therapeutic strategies for LCV are predominantly symptomatic, with an emphasis on conservative measures such as rest-limiting prolonged standing or ambulation and low-dose corticosteroids. Colchicine and various other unvalidated therapeutic options may also be considered, especially in cases where the condition is confined to the skin [5]. When LCV is drug-induced, the prognosis is generally favorable, with discontinuation of the offending medication leading to resolution. However, in instances where systemic vasculitis underpins the LCV, a more aggressive treatment regimen is warranted, typically involving higher doses of corticosteroids or immunosuppressive agents, tailored to the extent of organ involvement and the nature of the associated disease [5].

In most cases, first-line therapy is conservative, as the condition is often self-limiting. Initial recommendations include compression stockings and corticosteroid creams, along with options such as leg elevation and oral antihistamines. For clinically severe rashes or those unresponsive to conservative treatment, systemic prednisone at 40-60 mg daily can be initiated, with a tapering schedule over four to six weeks. In cases where prednisone is ineffective, as seen with our patient, adjunctive therapies like dapsone (100-200 mg) or colchicine (0.6 mg, two to three times daily) can be added. However, to reduce the risk of hemolytic anemia, especially in patients with G6PD deficiency, a G6PD level should be checked before starting these treatments. If the vasculitis remains unresponsive, additional immunosuppressive options, such as rituximab, mycophenolate mofetil, or methotrexate, may be considered [4].

## Conclusions

The idiopathic nature of LCV in this case presents a novel and compelling challenge in both clinical practice and research. While many cases of LCV are linked to identifiable triggers - such as infections, medications, or systemic diseases - idiopathic LCV, where no clear cause is established, adds an extra layer of complexity that warrants deeper exploration.

Reporting on idiopathic LCV is particularly valuable because it underscores the limits of our current understanding of vasculitic processes. Unlike LCV with known etiologies, idiopathic cases do not provide a direct path toward treatment, making treatment reliant on more trial-and-error methods. Moreover, idiopathic cases emphasize the importance of a tailored, patient-specific approach. Without the guidance of an underlying etiology, clinicians must prioritize detailed patient histories, thorough diagnostic workups, and careful symptom monitoring. Each idiopathic case that is reported enriches the literature by providing a deeper pool of data for clinicians and researchers to draw from. We hope to contribute our treatment success story to the scientific community and to provide not only a clinical guide but also a prompt for further research into this enigmatic and elusive condition.
